# New Locked-Wire-Type External Fixator (the Ichi-Fixator) for Fourth and Fifth Carpometacarpal Joint Dislocation

**DOI:** 10.1155/2018/8515781

**Published:** 2018-12-13

**Authors:** Satoshi Ichihara, Masao Suzuki, Akira Hara, Toshiya Kudo, Yuichiro Maruyama

**Affiliations:** ^1^Hand Surgery Center, Juntendo University Urayasu Hospital, Chiba, Japan; ^2^Department of Orthopedic Surgery, Juntendo University Urayasu Hospital, Chiba, Japan

## Abstract

We developed a new fixation method that involves the insertion of two wires and external wire fixation using a metal clamp. The aim of this technique was to increase the stability and rigidity of conventional percutaneous Kirchner wire fixation. Here, we present a patient with dislocation of the fourth and fifth carpometacarpal joints who was satisfactorily treated with closed reduction and percutaneous fixation with a linking external wire fixator (Ichi-Fixator). Operative treatment using the Ichi-Fixator system facilitates anatomical reduction and immediate full mobilization, resulting in good outcomes. The patient could perform all routine activities with normal grip strength and a full range of hand motion without pain. Such a treatment that improves comfort after the operation and may allow an immediate return to work will clearly boost patient satisfaction. Linked external wire-type fixation enables enhanced security of fixation, facilitates postoperative mobilization, and may allow an immediate return to work.

## 1. Introduction

Dislocation of the fourth and fifth carpometacarpal (CMC) joints can be managed conservatively with good outcomes if diagnosed early. However, if the initial reduction is unstable, it may be necessary to use Kirchner wire (K-wire) for fixation after closed or open reduction. Moreover, K-wire fixation can become displaced during early range-of-motion exercises. Consequently, the addition of a cast and splint after K-wire fixation may become necessary because of the fragility and instability of the fixation. The treatment options for CMC dislocation injuries include conservative management, closed reduction with K-wire fixation, and open reduction with internal fixation. Different treatment options have been described, but no clear consensus on the management of CMC dislocation injuries has yet emerged [1]. Furthermore, when left untreated, or if the reduction is incomplete, such injuries can lead to joint instability, early joint degeneration, chronic pain, stiffness, and posttraumatic arthritis. Patients are therefore at risk of long-term disability with osteoarthritis [2]. To help overcome these difficulties, we developed a new fixation method that involves the insertion of two wires and external wire fixation using a metal clamp. Two wires are secured together with special adjustable metal clamp fixation with small two screws. Because of the adjustable function of the small screws inside the fixator, the Ichi-Fixator enables to modulate under fluoroscopic inspection such as a static or distraction fixator. The aim of using this technique is to increase the stability and rigidity of conventional percutaneous K-wire fixation. Here, we present a patient with dislocation of the fourth and fifth CMC joints who was satisfactorily treated with closed reduction and percutaneous fixation with linking external wire fixation.

## 2. Case Report

**Figure 1:**
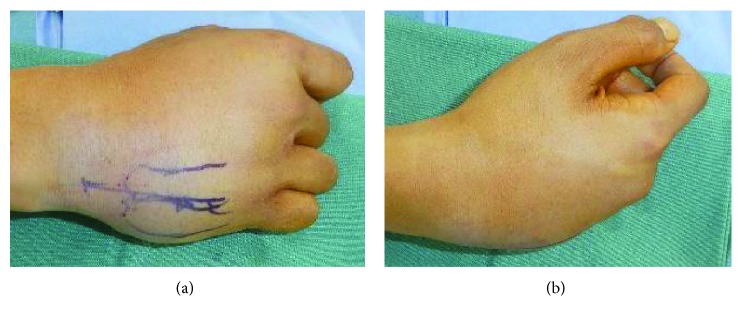
Apparent shortening of the fourth and fifth rays. (a) Mild swelling of the fourth and fifth CMC joint region, and (b) a bony prominence was felt dorsally.

**Figure 2:**
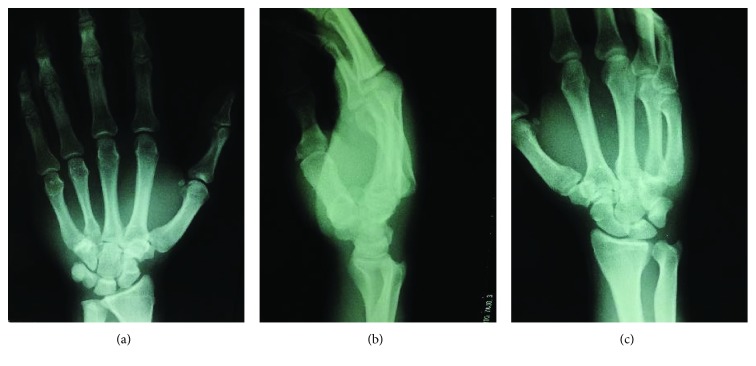
Anteroposterior (a), lateral (b), and internal oblique (c) radiographs of the right hand showed dorsal dislocation of the fourth and fifth CMC joints, without fracture.

A 27-year-old man presented with severe pain on the right carpus following a fall with injury to the right hand. There was mild swelling of the fourth and fifth CMC joint region, and a bony prominence was felt dorsally. Furthermore, there was apparent shortening of the fourth and fifth rays (Figures 1(a) and 1(b)). A diagnosis of the fourth and fifth CMC joint dislocation was made based on radiographs and computed tomography scans. Anteroposterior, lateral, and internal oblique radiographs of the right hand showed dorsal dislocation of the fourth and fifth CMC joints, without fracture (Figures 2(a)–2(c)). Immediate closed reduction was done in the operating room under locoregional anaesthesia by applying longitudinal traction and direct pressure on the metacarpal base dorsally. However, the achieved CMC joint reduction was unstable and easily dislocated dorsally on passive flexion of the metacarpal joint. Finally, we decided to perform external fixation using an Ichi-Fixator (Neo-medical, Saitama, Japan) linked-wire external fixator. Before the operation, the quick disabilities of the arm, shoulder, and hand questionnaire (QDASH) score were 52.95 and the visual analogue scale (VAS) pain score was 5/10.

### 2.1. Surgical Technique

**Figure 3:**
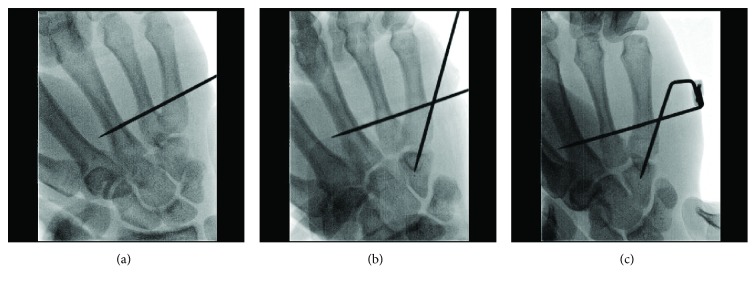
A 1.5 mm fixator pin was inserted from the base of the fifth metacarpal to the third metacarpal transversally (a). Another 1.5 mm fixator pin was inserted from the ulnar base of the fifth metacarpal to the hamate bone obliquely (b). The external ends of the two pins were bent so that they became parallel. The parallel ends of the two pins were then inserted in a metal clamp from the opposite sides (c).

**Figure 4:**
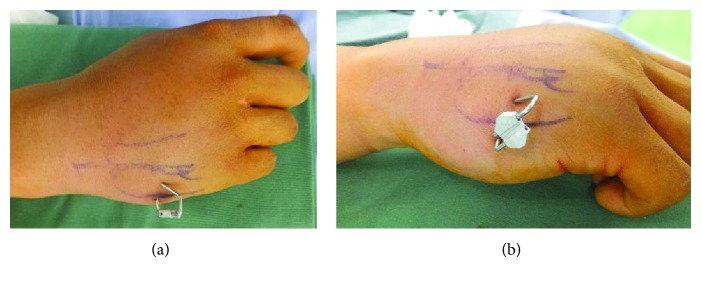
After fluoroscopic assessment, the ends of the two pins were fixed firmly together using a clamper. A true dorsal view (a) and an ulnar oblique view (b) of the right hand are shown.

**Figure 5:**
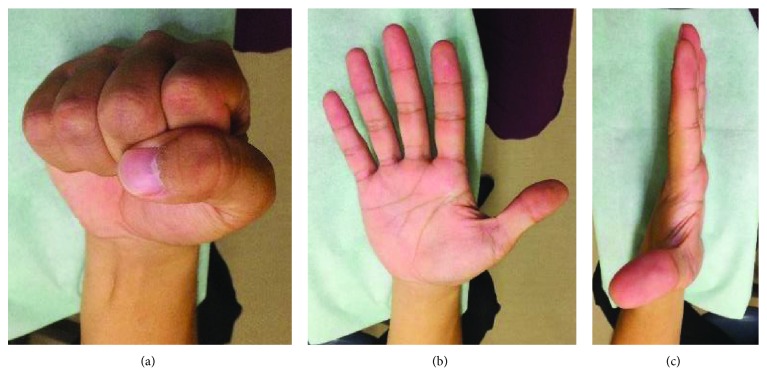
Total extension (a) and total flexion (b) of the right hand on a true dorsal view and total extension of the right hand on an ulnolateral view (c).

After closed reduction, a 1.5 mm fixator pin was inserted from the base of the fifth metacarpal to the third metacarpal transversally (Figure 3(a)). Another 1.5 mm fixator pin was inserted from the ulnar base of the fifth metacarpal to the hamate bone obliquely (Figure 3(b)). The external ends of the two pins were bent so that they became parallel. The parallel ends of the two pins were then inserted in a metal clamp from opposite sides (Figure 3(c)). After fluoroscopic assessment, the ends of the two pins were fixed firmly together using a clamper (Figures 4(a) and 4(b)). A light dressing was applied, and the patient was allowed to start range-of-motion exercises without immobilization immediately after the operation. The patient could use the hand for light activities just after the operation and could return to his regular work 3 days after the operation. The pins were removed in an outpatient setting when union of the fracture was verified radiologically. In the current case, the linked-wire external fixator was in place for 6 weeks. At the most recent follow-up, the QDASH score was 0.00 and the VAS score was 0/10. The grip strength and total active motion were 102% and 101%, respectively, compared with the contralateral side (Figures 5(a)–5(c)). The patient could perform all routine activities with normal grip strength and a full range of hand motion without pain. The patient could carry out his previous work just as before. He showed no pain and returned to all his previous activities without discomfort.

## 3. Discussion

CMC dislocations associated with fifth or other metacarpal fractures or hamate bone fractures are more frequent than pure dislocations. Pure dislocations of the fourth and fifth CMC joints without fractures are relatively rare injuries [3]. In one study of 20 cases, 30% of dislocations involved the second through fifth metacarpals, 30% involved the fourth and fifth metacarpals, and 25% represented an isolated fifth metacarpal dislocation [2]. In the study by Cobb et al., the K-wire fixation group returned to light activity work at an average of 7 weeks. In this group, grip strength was diminished by an average of 14 kg. Compared with these results, the use of the linked-wire external fixator enabled our patient to return to work immediately after the operation and to recover full grip strength. Whereas conventional K-wire fixation usually uses two wires to achieve cross pinning, the Ichi-Fixator system facilitates reliable two- or three-dimensional fixation. Such reliable fixation guards against secondary displacement after primary operation. Moreover, in contrast to conventional pinning, patients can start range-of-motion exercises immediately after the operation. In a previous study, locked-wire systems were found to better resist loosening [4, 5].

One of the advantages of the Ichi-Fixator system is anatomical fixation. Stability of the finger CMC joints is provided by a system of four ligaments: dorsal, multiple palmar, and two sets of interosseous ligaments (only one between the third and fourth metacarpals) [6]. At the base of the fourth and fifth metacarpals, intermetacarpal wire insertion mimics the attachment of intermetacarpal ligaments. Furthermore, oblique wire insertion from the base of the fifth metacarpal to the hamate bone augments the dorsal CMC ligament to protect against dorsal and ulnar dislocation. Finally, locking the two wires enables fixation as a rigid two- or three-dimensional structure. Another advantage of the Ichi-Fixator system is patient comfort after the operation. Patients do not need to guard against or fear K-wires loosening or becoming detached. It may be that the linkage of K-wires will allow the omission of all additional external splintage. This should minimize joint stiffness and the consequent need for hand therapy. Better functional outcomes might be achieved by early mobilization and conservative management, despite poorer anatomical restoration [7]. One literature review suggested that operative management is required to treat CMC joint injuries [8–11]. The need for anatomical reduction is not certain, but good outcomes have been achieved with treatment strategies that aim for anatomical restoration. The disadvantages of conventional wire fixation include limited stability, accidental removal, migration, wire fracture, and infection. In contrast, operative treatment using a linked-wire external fixator system facilitates anatomical reduction and immediate full mobilization, resulting in good outcomes. After anatomical reduction, the Ichi-Fixator system provides relatively solid fixation and allows early mobilization. In the present case, the patient could return to his previous work (desk work with the use of a computer) 1 day after the operation. After 14 days, the patient had almost fully recovered and was able to perform all required tasks at work. Such a treatment that improves comfort after the operation and may allow an immediate return to work will clearly boost patient satisfaction.

## 4. Conclusions

We report a case of dislocation of the fourth and fifth CMC joints successfully managed operatively with fixation using the new Ichi-Fixator connected wire system. Linked external wire-type fixation enables enhanced security of fixation, facilitates postoperative mobilization, and may allow an immediate return to work.

## Supplementary Materials

Supplementary Materials Total extension and total flexion of the right hand on a true dorsal view and total extension of the right hand on an ulnolateral view at final follow-up period.
